# A CGG Repeat Expansion in *CSNK1E* Associated with Progressive Myoclonic Epilepsy with Incomplete Penetrance

**DOI:** 10.1002/mds.30326

**Published:** 2025-08-01

**Authors:** Fulya Akçimen, Pilar Alvarez Jerez, Ulviyya Guliyeva, Jasmine Lee, Laksh Malik, Breeana Baker, Kamran Salayev, Sughra Guliyeva, Kimberley J. Billingsley, Henry Houlden, Andrew B. Singleton, Cornelis Blauwendraat, Sara Bandres‐Ciga, Rauan Kaiyrzhanov

**Affiliations:** ^1^ Molecular Genetics Section, Laboratory of Neurogenetics National Institute on Aging, National Institutes of Health Bethesda Maryland USA; ^2^ Center for Alzheimer's and Related Dementias National Institute on Aging Bethesda Maryland USA; ^3^ Department of Neurodegenerative Disease, UCL Queen Square Institute of Neurology University College London London UK; ^4^ MediClub Hospital Baku Azerbaijan; ^5^ Department of Neuromuscular Diseases, UCL Queen Square Institute of Neurology University College London London UK

**Keywords:** progressive myoclonic epilepsy, *CSNK1E*, short tandem repeats, incomplete penetrance

## Abstract

**Background:**

Progressive myoclonic epilepsy is a heterogeneous neurodegenerative disorder characterized by early‐onset myoclonus, epilepsy, generalized tonic–clonic seizures, and progressive neurological deterioration. Recently, a CGG repeat expansion and increased *CSNK1E* DNA methylation have been shown to be associated with developmental and epileptic encephalopathies.

**Objective:**

To identify structural variants or repeat expansions associated with progressive myoclonic epilepsy in an Azerbaijani family using long‐read sequencing.

**Methods:**

Known genetic causes of progressive myoclonic epilepsy were ruled out through quadro‐exome sequencing in an individual exhibiting tonic–clonic seizures, dementia, and cerebellar ataxia with an age at onset of 10 years. After ruling out the presence of any other pathogenic mutation, long‐read whole genome sequencing was performed to investigate structural variants or repeat expansions potentially associated with the disease.

**Results:**

We identified a heterozygous expanded (CGG)_n_ repeat in exon 1 of *CSNK1E* in the proband (longest repeat length, n = 745) and her unaffected sister (longest repeat length, n = 980). The unaffected father was wild‐type, while the unaffected mother had an intermediate‐sized repeat expansion (n = 131), which might have expanded to a pathogenic length in the siblings upon transmission. The expanded allele exhibited higher methylation levels than the wild‐type, with globally elevated methylation in both siblings compared with parental samples.

**Conclusions:**

We suggest the association of the *CSNK1E*‐CGG expansion with incomplete penetrance in an Azerbaijani case with progressive myoclonic epilepsy, broadening its phenotypic spectrum. Our findings support the utility of long‐read sequencing and methylation analysis as powerful approaches to identifying and characterizing disease‐associated expanded repeats. © 2025 The Author(s). *Movement Disorders* published by Wiley Periodicals LLC on behalf of International Parkinson and Movement Disorder Society.

Progressive myoclonus epilepsies (EPMs) are rare neurodegenerative diseases characterized by myoclonus, epilepsy, and progressive cognitive impairment, sometimes accompanied by ataxia, intention tremor, and dysarthria^1^, ^2^. EPMs are clinically and genetically heterogeneous. Biallelic missense, frameshift, short insertions, and deletion variants in *CSTB* (MIM:254800),^3^ *PRICKLE1* (MIM:612437),^4^ *NHLRC1* (MIM:620681),^5^ *SCARB2* (MIM:254900),^6^ *KCTD7* (MIM:611726),^7^ *GOSR2* (MIM:614018),^8^ *CERS1* (MIM:616230),^9^ *LMNB2* (MIM:616540),^10^ *PRDM8* (MIM:616640),^11^ and *SLC7A6OS* (MIM:619191)^12^ are associated with recessive forms of EPM, while de novo heterozygous variants in *KCNC1* (MIM:616187)^13^ and *SEMA6B* (MIM:618876)^14^ have also been reported. In addition, biallelic dodecamer repeat expansions in the promoter of *CSTB* cause autosomal recessive EPM type 1A.^15^

To date, more than 10 CCG/CGG disease‐causing repeats in known fragile sites have been shown to cause diverse neurological diseases, including fragile X syndrome (*FMR1*), neurodevelopmental disorder with growth retardation (*FRA10AC1*), and intellectual developmental disorder (*DIP2B* in FRA12A site).^16^ These fragile sites show aberrant CpG methylation upstream and at the repeat region. Hypermethylated CGG short tandem repeats are usually positioned within a gene or its promoter region and cause hyper‐condensation, hindering the cellular transcription machinery from accessing essential binding sites and, consequently, leading to gene silencing.^17^, ^18^, ^19^

Long‐read whole genome sequencing approaches have been shown to be effective in identifying disease‐causing short tandem repeat expansions.^20^ The read length attained using Oxford Nanopore Technologies (ONT) enables the characterization of these expansions. The recent identification of GC‐rich repeats in *NOTCH2NL*,^21^ *NUTM2B‐AS1*,^22^ and *ZFHX3* ^23^, ^24^ motivated the search for expanded repeats in neurological phenotypes with no definitive causal variants.

## 1 Methods

### 1.1 Study Participants

**FIG. 1:**
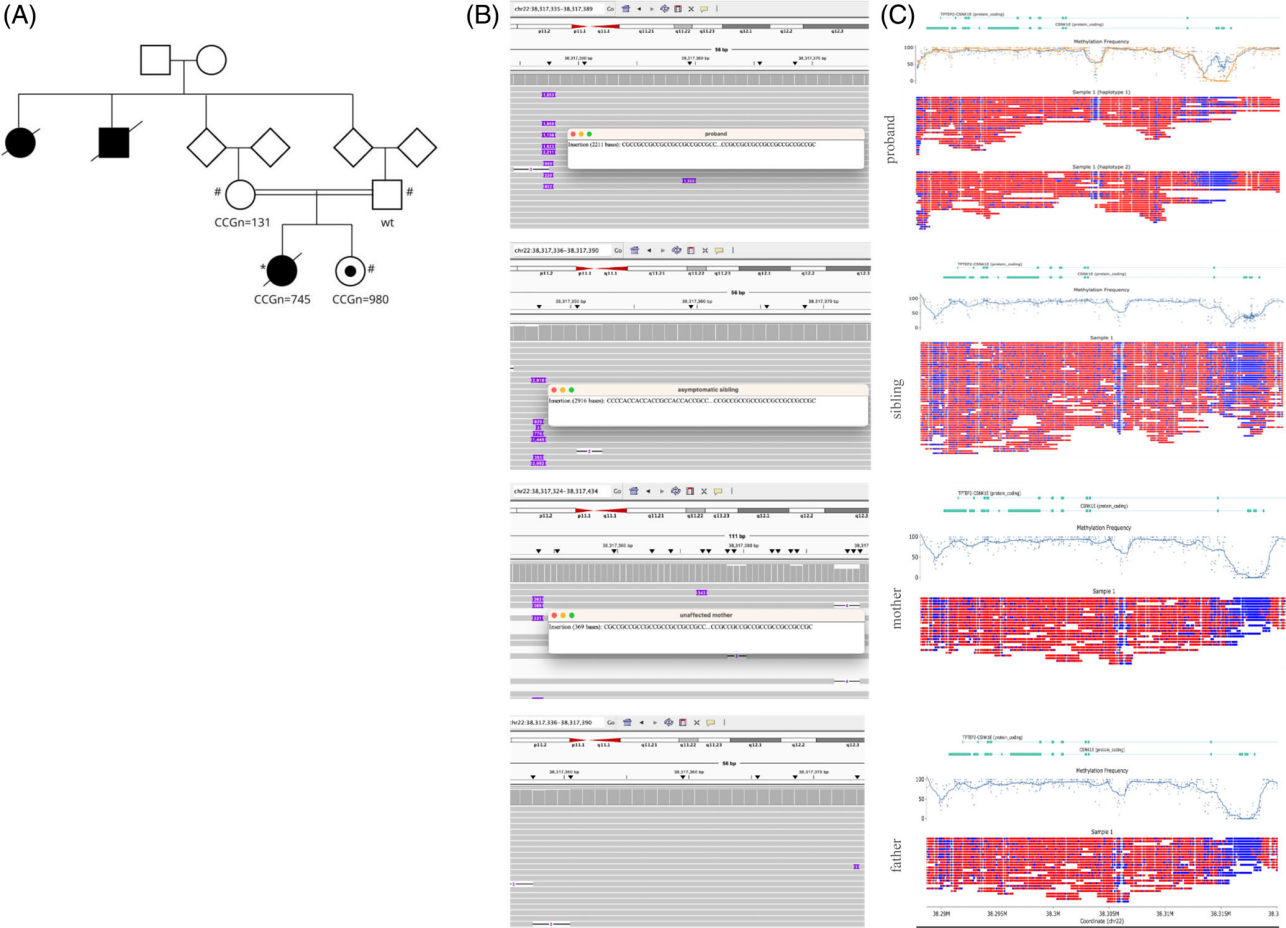
(A) Family pedigree. Parents are first‐degree cousins. *ONT (Oxford Nanopore Technologies) whole genome long‐read sequencing, ^#^adaptive ONT long‐read sequencing. (B) Integrative genome viewer image of short tandem repeat region in intron 1 of *CSNK1E* (GRCh38 is the reference genome). (C) Methylation plots were generated by Modbamtools. We were only able to phase the proband's ONT long‐read sequencing data, which showed higher methylation in the allele with expansion (blue shows the reads with expansion, whereas orange shows wild‐type). [Color figure can be viewed at wileyonlinelibrary.com]

An Azerbaijani family, including the proband with EPM, her unaffected sister, and parents, were recruited to the Synaptopathies and Paroxysmal Syndromes (SYNaPS) Study in 2019, held by the Institute of Neurology University College London (IoN UCL) (Fig. 1A). The institutional review boards of IoN UCL as well as the respective Azerbaijani institution approved this study (07/Q0512/26). Written informed consent was obtained from each participant.

### 1.2 Exome Sequencing

DNA was extracted from peripheral blood samples according to standard procedures. Quadro‐exome sequencing (ES) was performed in the proband, parents, and unaffected sibling as described elsewhere,^25^ and our bioinformatics filtering strategy included screening for only protein‐altering and donor/acceptor splicing variants. In accordance with the pedigree and phenotype, priority was given to rare variants (<0.5% in public databases, including the Genome Aggregation Database [gnomAD] v4, UK Biobank, Genomics England) that were fitting a recessive (homozygous or compound heterozygous) or a de novo model and/or variants in genes previously linked to epilepsy progressive myoclonus, epilepsy, cerebellar ataxia, movement disorders and other neurodevelopmental and neurodegenerative disorders in OMIM. Utilizing gnomAD v4.1 (https://gnomad.broadinstitute.org/), CADD v.1.7 (https://cadd.gs.washington.edu/), and OMIM annotations (genemap2, downloaded from omim.org in September 2024), we evaluated all rare protein‐altering variants identified in the proband, prioritizing those with a CADD>12.36 that could be associated with the phenotype.

### 1.3 ONT Long‐Read Whole Genome Sequencing, Variant Calling, and Annotation

Long‐read library preparation and whole genome sequencing for the proband were performed as previously described.^26^, ^27^ In summary, libraries were prepared using ONT's LSK114 kit, and the sample was sequenced on a PromethION R10.4.1 flow cell (ONT, Oxford, UK) for 72 hours. Base calling was performed with Guppy version 6.3.8^28^ with the super‐accuracy model, preserving methylation tags with the –bam‐out option, and alignment to the GRCh38 reference genome was done using minimap2 version 2.26.^29^

Germline variant calling was performed using Clair3 version 1.0.4^30^ and structural variants were called using Sniffles2 version 2.3.3,^31^ all with default parameters. To examine short tandem repeat expansions, we performed an alignment using LAST version 1544^32^ and variant calling using tandem‐genotypes package version 1.9.1.^33^ Missense and short insertion and deletion variants were annotated using ANNOVAR^34^ (version date 2020‐06‐08). Large structural variants were annotated by AnnotSV version 3.3.7.^35^ Utilizing structural variant type and OMIM annotations, we evaluated all large deletions, insertions, and known repeat expansions that could be associated with the phenotype.

A targeted long‐read sequencing approach with adaptive sampling was employed on the ONT platform to examine the *CSNK1E*‐CGG expansion in the proband's younger sister and parents. Libraries were prepared using the ONT's SQK‐NBD114.24 kit for each sample. The barcoded samples were pooled equimolar and 50 fmol was loaded onto a PromethION R10.4.1 flow cell. A custom BED file covering the region of interest at chr 22:25,500,001 – 48,100,001 (GRCh38) was used for adaptive sampling. Base calling and demultiplexing were performed by Dorado v7.2.13 in MinKNOW (v23.11.4) with the high accuracy basecalling model including modified basecalling for 5mC and 5hmC in CG contexts. See the full protocol at https://www.protocols.io/view/native‐barcoding‐sqk‐nbd114‐gdna‐for‐adaptive‐samp‐kxygx3qx4g8j/v1.

### 1.4 Methylation Analysis

Haplotype phasing of long reads was done using PEPPER deepvariant tool version 0.8.^36^ Haplotype‐specific methylation plots were generated using modbamtools version 0.4.8^37^ with the Gencode v.38 GRCh38 gene transfer format file as annotation (https://www.gencodegenes.org/human/release_38.html). Phased bams and their methylation tags were also visualized on the Integrative Genomics Viewer version 2.16.0.^38^

### 1.5 Characterization of the *CSNK1E* Short Tandem Repeats in 1000 Genomes ONT Cohort

We downloaded 908 hg38‐aligned CRAM files from the 1000 Genomes Project (1KGP) ONT panel dataset. Basecalling and alignment pipeline are described at https://ftp.1000genomes.ebi.ac.uk/vol1/ftp/data_collections/1KG_ONT_VIENNA/README_1KG_ONT_VIENNA_20240227.md. We first extracted the *CSNK1E* repeat region and converted it to fastq format using SAMtools version 1.19.^39^ Extracted reads were aligned using LAST version 1544,^40^ and genotyped repeats using tandem‐genotypes version 1.9.1.^33^ The difference in repeat length among five super‐populations was compared using one‐way ANOVA with Tukey's post hoc test.

## 2 Results

### 2.1 Clinical Characterization

The proband is a 23‐year‐old female, born at full term after an uncomplicated pregnancy to a consanguineous family (first cousins) of Azerbaijani ancestry (Figure 1A). Her early development was normal, but she began experiencing myoclonic episodes at the age of 10 years. A year later, she developed tonic–clonic seizures and currently suffers from daily generalized tonic–clonic seizures (GTCs). The myoclonic jerks can occur at any time but are more pronounced upon awakening. These jerks appear in bursts, affecting both axial muscles and hands. She has been experiencing cognitive decline since the age of 12 years. The patient frequently reported experiencing spontaneous flashing before her eyes. Neurological regression began around the same time, with a loss of speech and difficulty swallowing by the age of 21 years, without any apparent trigger. Although her feeding difficulties gradually improved over 5 months, allowing her to regain some speech, she started exhibiting progressive gait instability, eventually losing the ability to walk independently by the age of 21 years. Starting at the age of 14 years, she began experiencing urinary incontinence.

A computed tomography scan revealed epiphyseal calcifications and calcifications of the interhemispheric falx. An electroencephalogram (EEG) showed frequent bilateral synchronous spike–wave activity, predominantly in the frontal region (Figure S1). However, not all myoclonic jerks were associated with spike‐polyspike wave activity on the EEG. Fundoscopic examinations were unremarkable. Despite being on primidone, zonisamide, and clobazam, she continues to have frequent daily seizures, including up to 10 generalized tonic–clonic seizures and frequent myoclonic jerks, which are often triggered by anxiety or movement. Other antiseizure medications have been tried: valproic acid caused excessive sleepiness, and levetiracetam and clonazepam were not beneficial. The proband passed away recently at the age of 23 years.

Her 18‐year‐old unaffected sister has experienced sensations of jerking in her shoulders since the age of 12 years. However, no visible jerks or other types of seizures have been observed, and her neurological examination is unremarkable. The parents share a common uncle and aunt (siblings) who both had epilepsy characterized by myoclonic jerks and progressive developmental regression, ultimately resulting in death during the second decade of life.

### 2.2 Identification of a *CSNK1E* CGG Repeat Expansion

First, short‐read proband exome sequencing (ES) was performed to exclude rare variants in known EPM‐associated genes that could explain the proband's phenotype, including tonic–clonic seizures, dementia, and cognitive deterioration. Then, rare single nucleotide variants, as well as small deletions and insertions in candidate novel disease genes, were explored. No rare, potentially pathogenic variants linked to EPM or neurodevelopmental diseases were identified. ES in the family did not reveal any clinically relevant variants that could explain the phenotype of the proband (Tables S1 and S2). Next, we examined large structural variants and short tandem repeat expansions using ONT long‐read sequencing data. From the whole genome data, we identified a heterozygous expanded CGG tandem repeat in exon 1 of *CSNK1E* (chr22:38,317,282‐38,317,375) (Figure 1B, Table S3). The tandem‐genotype package determined the longest expanded allele as CGG_n_ = 745 in the proband, CGG_n_ = 131 in the unaffected mother, CGG_n_ = 8 in the unaffected father, and CGG_n_ = 980 in the unaffected sibling. We did not identify any other structural variants in a gene associated with epilepsy, neurodegenerative, or neurodevelopmental disease that could explain the disease phenotype. Although we identified a total of 772 large structural variants, 82 of them were longer than 1 kb. No other large insertions, deletions, or repeat expansions that could explain the phenotype in the proband were identified (Supplementary Table S3).

### 2.3 Hypermethylation is Observed in the CGG‐Expanded Allele

After phasing of long‐read data, we examined the allele‐specific 5‐mCpG methylation status in the repeat sequence for the proband. The expanded allele showed higher methylation compared with the wild‐type, likely due to the addition of CpG sites from the repeat motif. Phasing of the proband's family from the adaptive sampling data was not successful, so methylation was inspected globally and not by haplotype. This global methylation analysis in the family members identified elevated methylation levels around the repeat sequence in the proband and unaffected sibling compared with the unaffected parents. No methylation differences were observed between the mother and father (Figure 1C).

### 2.4 *CSNK1E* Short Tandem Repeat Length and Motifs in 1000 Genomes ONT Cohort

To examine the distribution of the repeat length in the general population, we utilized the 1KGP ONT panel dataset. The dataset included 908 participants (245 African, 151 American, 17s7 East Asian, 166 European, and 169 South Asian ancestry individuals). A total of 98.7% of the participants had a *CSNK1E* repeat length of less than 20 in the general population. The longest *CSNK1E* repeat expansions were identified in participants of South Asian (CGG_n_ = 48) and European ancestries (CGG_n_ = 47) (Figure S2). Furthermore, we did not observe any differences among the five studied super‐populations (one‐way ANOVA, *P* = 0.154).

## 3 Discussion

We describe a long CGG expansion (CGG_n_ = 745) in the 5′‐UTR of *CSNK1E* at the fragile FRA22A site as a possible cause of progressive myoclonic epilepsy with ataxia and progressive cognitive deterioration. The proband and her younger sibling were positive for the expanded repeat; however, the younger sibling (18 years old) did not exhibit the phenotype. The parents were first cousins; while the unaffected father was wild‐type for the expansion, the unaffected mother had an intermediate‐size repeat expansion, which might have enlarged into a pathogenic expansion in the siblings upon transmission.

A recent study utilizing methylation analysis and long‐read sequencing identified a *CSNK1E*‐CGG repeat expansion in three individuals with pediatric epileptic encephalopathy, where other potential causal mutations were excluded. The hypermethylation and expansion were also observed in some of the unaffected relatives, highlighting its incomplete penetrance. Our family, which includes two carriers and one proband affected by EPM, provides additional evidence supporting the incomplete penetrance of the *CSNK1E* repeat expansion.^41^

The proband in our study exhibited a different phenotype compared with the previously reported three cases, who were diagnosed between 9 months and 4–5 years of age and presented with developmental and epileptic encephalopathy (DEE). Although the proband in this report and the previously described cases shared a common feature of epileptic disorders, there were significant differences in the age of disease onset, phenotype spectrum, and severity.^41^ Unlike the previously reported cases, our proband did not exhibit developmental abnormalities and presented with a phenotype suggestive of EPM rather than DEE.

Interestingly, mothers of two previously reported cases were found to carry the *CSNK1E* CGG repeat expansion: one remained unaffected, while the other had neurodevelopmental, cognitive, and sleep‐related symptoms but no seizure history. Her difficulties included dyslexia and learning impairments necessitating placement in a special needs classroom from fourth grade onward.^41^ Notably, a recent multi‐ancestry population‐based study demonstrated that the frequency of disease‐associated pathogenic repeats is higher than previously estimated in clinical studies, reinforcing the incomplete penetrance of certain repeat expansions.^42^

Our study has some limitations. Although similar phenotypes were observed in the parent's aunt and uncle, both individuals passed away during the second decade of life without receiving a diagnosis, limiting the ability to confirm the genetic basis of their symptoms. Their clinical similarity to the proband raises the possibility that they were carriers of the same repeat expansion. Given that the parents are first cousins and share this maternal aunt and uncle, it is likely that the expansion was inherited through a shared ancestral haplotype. However, in the absence of biological samples or detailed clinical documentation, it remains uncertain whether their phenotype was fully consistent with that of the proband.

Another limitation is the inability to resolve methylation status by haplotype. Although adaptive sampling was performed, the available read lengths were insufficient for phasing, precluding allele‐specific methylation analysis. Nevertheless, we identified elevated global methylation levels around the *CSNK1E* repeat sequence in the proband and sibling compared with the parents.

Characterizing the frequency of this repeat expansion among Azerbaijani individuals and additional diverse populations is crucial to understanding its potential role in EPM. However, the pathogenic expansion spans more than 745 CGG units (over 2000 base pairs), making short‐read sequencing‐based biobanks ineffective for its detection. Although long‐read sequencing efforts have been initiated in some biobanks, most include only adult participants, except for Genomics England (https://www.genomicsengland.co.uk/). Nevertheless, even in such initiatives, the number of long‐read sequenced samples remains insufficient to evaluate a repeat expansion associated with a disease of such low prevalence.

In conclusion, we suggest the association of the *CSNK1E*‐CGG expansion with incomplete penetrance in an Azerbaijani family. Overall, our findings underscore the critical role of long‐read sequencing in accurately identifying and characterizing expanded repeats in complex neurological disorders. Future studies incorporating long‐read sequencing data and participants from underrepresented populations into biobanks will be pivotal in further understanding the full spectrum of phenotypic variability, and the penetrance.

## Author Roles

(1) Research Project: A. Conceptualization, B. Design, C. Data Acquisition, D. Sequencing, E. Genomic Analysis; (2) Statistical Analysis: A. Design, B. Execution, C. Review and Critique; (3) Manuscript Preparation: A. Writing of the First Draft, B. Review and Critique; (4) A. Project Supervision.F.A.: 1E, 3A, 3B

P.A.J.: 1E, 3A, 3B

U.G.: 1C, 3B.

J.L.: 1D, 3B.

L.M.:1C, 1D.

B.B.: 1C, 1D.

K.S.: 1C, 3B.

S.G.: 1C, 3B.

K.J.B.: 1C, 1D.

H.H.: 1A, 1B, 1C, 3B, 4A.

A.B.S.: 1A, 1B, 3B, 4A.

C.B.: 1A, 1B, 3B, 4A.

S.B.‐C.: 1A, 1B, 3B, 4A.

R.K.: 3A, 3B, 4A.

## Funding

This work was supported in part by the Intramural Research Programs of the National Institute on Aging (NIA) and the National Institute of Neurological Disorders and Stroke (NINDS), National Institutes of Health, Department of Health and Human Services (project numbers Z01‐AG000949 and 1ZIANS003154). Computational analyses were performed using the NIH HPC Biowulf cluster (http://hpc.nih.gov).

This study was funded by the Medical Research Council (MRC) (MR/S01165X/1, MR/S005021/1, G0601943, MR/V012177/1), The Wellcome Trust and strategic award (Synaptopathies) funding (WT093205 MA and WT104033AIA). For the purpose of Open Access, the author has applied a CC BY public copyright license to any Author Accepted Manuscript version arising from this submission.

## Supporting information


**Figure S1.** Electroencephalography (EEG) shows slow background activity in the theta range (7 Hz), with diffuse asynchronous spike–wave activity most prominent over the frontal region. Spikes are occasionally observed following rhythmic photic stimulation; however, there is no consistent correlation with the frequency of the stimulation.

**Figure S2.** Distribution of the repeat length in the 1KGP ONT (Oxford Nanopore Technologies) dataset. A total of 98.7% of the participants had a *CSNK1E* repeat length of less than 20 in the general population. The longest *CSNK1E* repeat expansions were identified in participants with South Asian (CGG_n_ = 48) and European ancestry (CGG_n_ = 47). No differences among the five super‐populations were observed (one‐way ANOVA, *P* = 0.154).

**Table S1.** Candidate protein‐altering variants identified in quadro‐exome sequencing.
**Table S2.** Candidate protein‐altering variants identified in quadro‐exome sequencing in the family after segregation analysis.
**Table S3.** Candidate structural variants identified in long‐read sequencing in the proband.

## Data Availability

Extracted DNA for 1000 Genomes Project was obtained from the Coriell Institute for Medical Research and was consented for the full public release of genomic data. See Coriell (https://www.coriell.org) for more information on specific cell lines. 1000 Genomes Project ONT dataset was generated at the Institute of Molecular Pathology (Vienna, Austria) with funds provided by Boehringer‐Ingelheim. Long‐read sequencing data generated during the current study are not publicly available due to privacy/ethical restrictions.
