# Supplementary figures and images for: A CGG Repeat Expansion in CSNK1E Associated with Progressive Myoclonic Epilepsy with Incomplete Penetrance
Source: Mov Disord. 2025 Aug 1;40(11):2469–75. doi: 10.1002/mds.30326 (PMC12661629; doi:10.1002/mds.30326)

## Slide 1
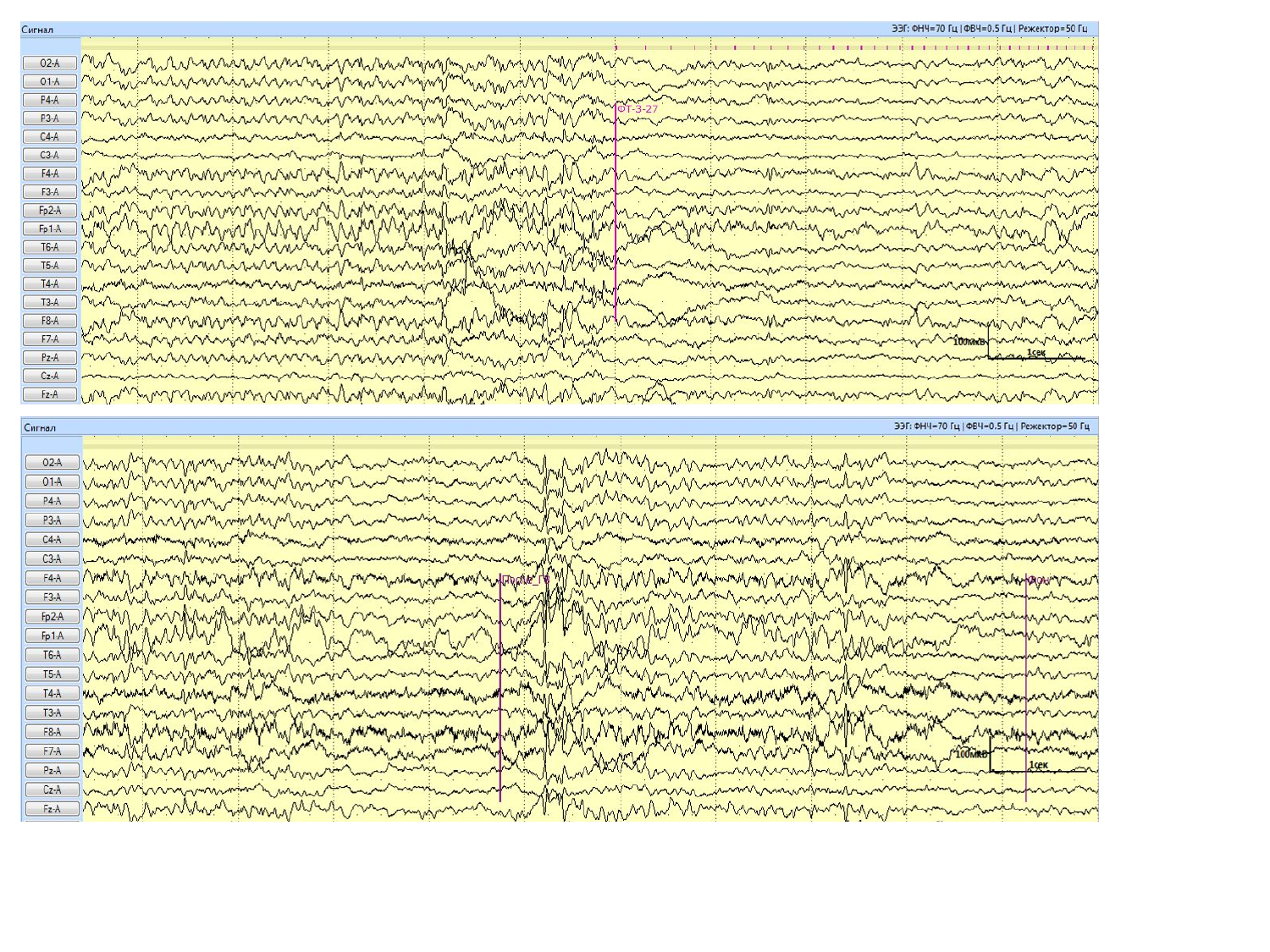

Supplement: Supplementary file 1 — Figure S1. Electroencephalography (EEG) shows slow background activity in the theta range (7 Hz), with diffuse asynchronous spike–wave activity most prominent over the frontal region. Spikes are occasionally observed following rhythmic photic stimulation; however, there is no consistent correlation with the frequency of the stimulation. [file MDS-40-2469-s002.pptx]

## Slide 1
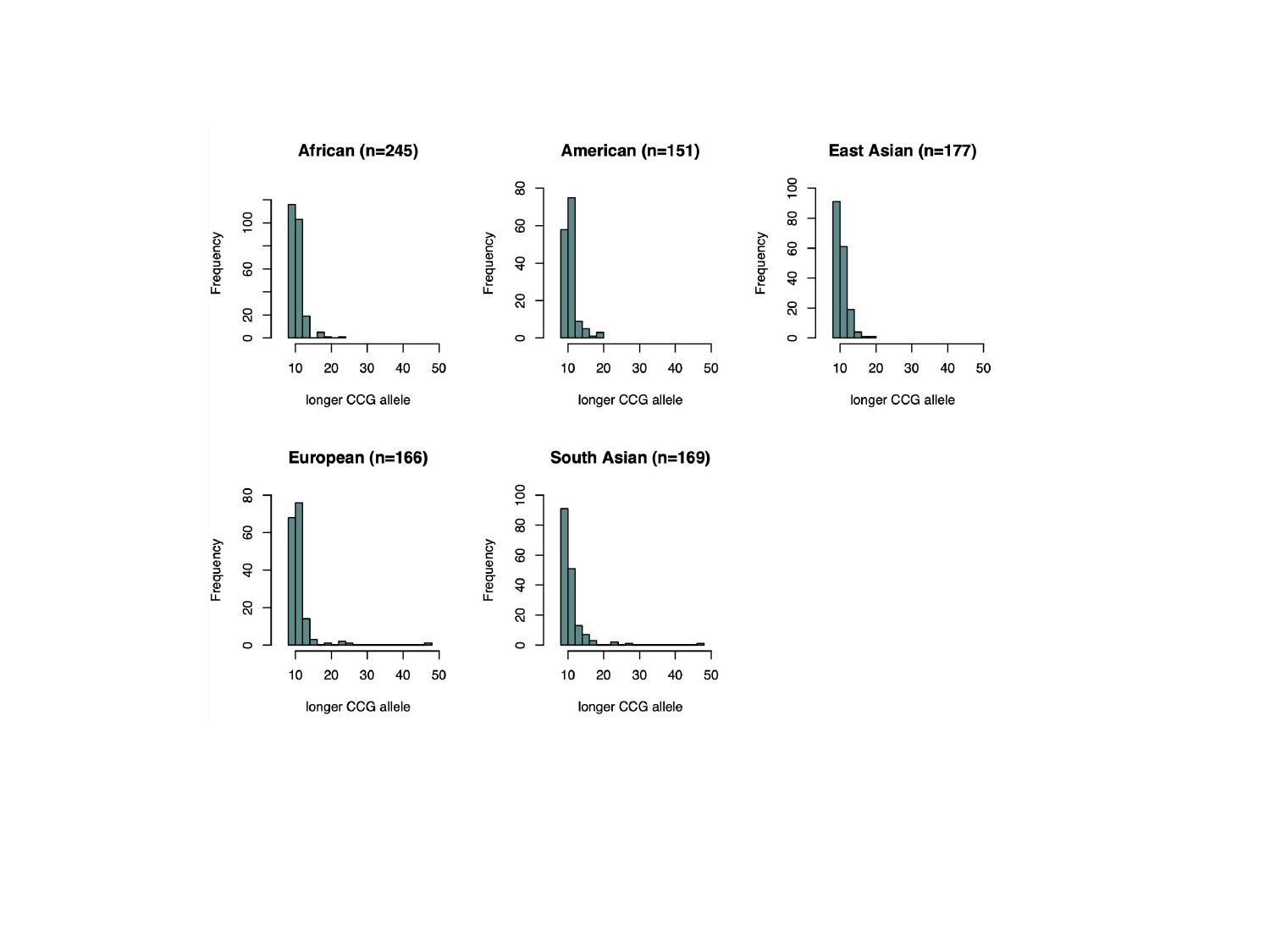

Supplement: Supplementary file 2 — Figure S2. Distribution of the repeat length in the 1KGP ONT (Oxford Nanopore Technologies) dataset. A total of 98.7% of the participants had a CSNK1E repeat length of less than 20 in the general population. The longest CSNK1E repeat expansions were identified in participants with South Asian (CGGn = 48) and European ancestry (CGGn = 47). No differences among the five super‐populations were observed (one‐way ANOVA, P = 0.154). [file MDS-40-2469-s003.pptx]
